# Antimicrobial nisin acts against saliva derived multi-species biofilms without cytotoxicity to human oral cells

**DOI:** 10.3389/fmicb.2015.00617

**Published:** 2015-06-18

**Authors:** Jae M. Shin, Islam Ateia, Jefrey R. Paulus, Hongrui Liu, J. Christopher Fenno, Alexander H. Rickard, Yvonne L. Kapila

**Affiliations:** ^1^Department of Periodontics and Oral Medicine, University of Michigan School of Dentistry, Ann ArborMI, USA; ^2^Department of Biologic and Materials Sciences, University of Michigan School of Dentistry, Ann ArborMI, USA; ^3^Department of Epidemiology, University of Michigan School of Public Health, Ann ArborMI, USA

**Keywords:** nisin, dental plaque, saliva derived multi-species biofilms, human oral cells, confocal, apoptosis, oral diseases

## Abstract

**Objectives:** Nisin is a lantibiotic widely used for the preservation of food and beverages. Recently, investigators have reported that nisin may have clinical applications for treating bacterial infections. The aim of this study was to investigate the effects of ultra pure food grade Nisin ZP (>95% purity) on taxonomically diverse bacteria common to the human oral cavity and saliva derived multi-species oral biofilms, and to discern the toxicity of nisin against human cells relevant to the oral cavity.

**Methods:** The minimum inhibitory concentrations and minimum bactericidal concentrations of taxonomically distinct oral bacteria were determined using agar and broth dilution methods. To assess the effects of nisin on biofilms, two model systems were utilized: a static and a controlled flow microfluidic system. Biofilms were inoculated with pooled human saliva and fed filter-sterilized saliva for 20–22 h at 37°C. Nisin effects on cellular apoptosis and proliferation were evaluated using acridine orange/ethidium bromide fluorescent nuclear staining and lactate dehydrogenase activity assays.

**Results:** Nisin inhibited planktonic growth of oral bacteria at low concentrations (2.5–50 μg/ml). Nisin also retarded development of multi-species biofilms at concentrations ≥1 μg/ml. Specifically, under biofilm model conditions, nisin interfered with biofilm development and reduced biofilm biomass and thickness in a dose-dependent manner. The treatment of pre-formed biofilms with nisin resulted in dose- and time-dependent disruption of the biofilm architecture along with decreased bacterial viability. Human cells relevant to the oral cavity were unaffected by the treatment of nisin at anti-biofilm concentrations and showed no signs of apoptotic changes unless treated with much higher concentrations (>200 μg/ml).

**Conclusion:** This work highlights the potential therapeutic value of high purity food grade nisin to inhibit the growth of oral bacteria and the development of biofilms relevant to oral diseases.

## Introduction

Dental plaque is an architecturally complex bacterial multi-species biofilm community (Marsh, 2010; Zijnge et al., 2010). Hundreds of species can coexist in these communities and together they can be up to 1000-times more resistant to antimicrobials than their planktonic counterparts (Gilbert et al., 2002; Aas et al., 2005). Enhanced antimicrobial resistance accounts, in part, for the accumulation of pathogens that are associated with dental caries, periodontal disease, and pulpal infections (Marsh, 2003). Strategies to control biofilms and their contained species have met with difficulties, as is evidenced by the public health burden associated with poor oral health. The Centers for Disease Control and Prevention (CDC) estimated that in the United States (US) alone, $108 billion was spent on dental services in 2010 and is therefore constantly on the search for alternative cost-effective preventative strategies (Chronic Disease Prevention and Health Promotion, 2015). Given the public health burden associated with dental plaque, new candidate anti-biofilm technologies are currently being investigated. These include modifications to traditional approaches, such as the development of improved antimicrobial compounds and formulations (zinc, cetylpyridinium chloride, stannous compounds, natural agents) (Allaker and Douglas, 2009; Marsh, 2010) to more innovative technologies, such as those that display antimicrobial but also anti-biofilm effects (Kaplan, 2010). For example, there has been considerable interest in approaches to inhibit cell–cell signaling between bacteria to control oral biofilm formation (Raffa et al., 2005; Barrios et al., 2006; Rickard et al., 2006; Bordi and de Bentzmann, 2011; Cuadra-Saenz et al., 2012) and to augment cell–cell signaling for enhancing antimicrobial activity (Eckert et al., 2006; Ahmed et al., 2009). In addition, there has been substantial interest in approaches to structurally weaken biofilms by targeting bacterially produced extracellular polymeric substances (EPS) using enzymes such as dispersin B (Izano et al., 2007). One technology that has recently garnered attention is the use of bacteriocins, such as nisin, which is produced by *Lactococcus lactis* (Pepperney and Chikindas, 2011; Arthur et al., 2014).

*Lactococcus lactis* is a non-pathogenic, Gram-positive, lactic acid bacterium used in food fermentation and found commonly in dairy products (De Vuyst and Vandamme, 1994; Cleveland et al., 2001). In addition, *L. lactis* has been proposed as a probiotic agent (Vinderola and Reinheimer, 2003). Studies have implied that lactic acid bacteria can prevent the co-localization of pathogenic bacteria in certain microflora (intestinal, vaginal) by stabilizing the complex biofilm community (Todorov et al., 2007; Kindrachuk et al., 2013). These processes may be mediated by bacteriocins like nisin. Nisin is a bacterially secreted polypeptide composed of 34-amino acids that exists as two natural variant forms; nisin A and Z. The two variants differ only by a single amino acid at position 27; histidine in nisin A and asparagine in nisin Z (Mulders et al., 1991). Nisin has amphipathic and cationic properties, and is classified as a Type A (I) lantibiotic (Gross and Morell, 1971; Asaduzzaman and Sonomoto, 2009). Lantibiotics like nisin are known for their broad-spectrum Gram-positive antimicrobial activities, high potency, low association with cytotoxicity, and lack of stable and transmissible antimicrobial resistance (Sahl and Bierbaum, 1998; Willey and van der Donk, 2007; Smith and Hillman, 2008). As a food preservative, nisin has been granted GRAS (Generally Regarded as Safe) status by the U.S. Food and Drug Administration (1988; FDA Federal Register, 1998) for use in pasteurized, processed cheese spreads and is currently licensed in 48 countries (Cotter et al., 2005). Nisin acts by altering the structure of the cellular membrane by forming short-lived pores and binding to the bacterial cell-wall precursor lipid II with high affinity (Breukink et al., 1999; Wiedemann et al., 2001). This initial interaction facilitates further modes of action to inhibit the cell wall biosynthesis, spore outgrowth, and activation of autolytic enzymes (Peschel and Sahl, 2006). Thus, the lack of bacterial resistance toward nisin likely stems from its interaction with a number of distinct targets.

Investigators from multiple fields have shown promising results for the use of nisin to treat bacterial infections, such as mastitis in humans and cows (Cao et al., 2007; Wu et al., 2007; Fernández et al., 2008), *Staphylococcus aureus* infections in atopic dermatitis (Valenta et al., 1996), respiratory tract infections (Bush and Macielag, 2000; De Kwaadsteniet et al., 2009), and experimental gingivitis in dogs (Howell et al., 1993). Recent *in vitro* and *in vivo* evidence has even indicated a role for nisin as an anticancer agent (Joo et al., 2012). Given the high therapeutic potential of nisin, we hypothesize that nisin can be utilized as an antimicrobial and anti-biofilm agent to prevent or treat oral biofilm associated diseases. Thus, the aim of this work was to determine the effectiveness of nisin in inhibiting the formation and maintenance of saliva derived multi-species oral biofilms. In addition, we examined the ability of nisin to inhibit the growth of common oral bacteria found in the human oral cavity, including both Gram-positive and Gram-negative, and aerobic and anaerobic bacteria. Lastly, we examined the potential cytotoxicity of nisin on human cells relevant to the oral cavity.

## Materials and Methods

### Nisin Preparation

An ultra pure food grade (>95%) form of the nisin Z, referred to here as nisin ZP, was purchased from Handary (S.A., Brussels, Belgium), a primary manufacturer of nisin in the food industry. From here forward, nisin ZP will be referred to as nisin. Nisin powder was stored at 4°C in a vacuum desiccator (Thermo Scientific, Waltham, MA, USA). The stock solution was prepared at a concentration of 5 mg/ml in water, filter sterilized, and stored at 4°C for a maximum of 5 days for use in experiments.

### Bacterial Strains and Growth Conditions

*Streptococcus oralis* 34 (kindly donated by P. E. Kolenbrander, National Institutes of Health, Bethesda, MA, USA), *Streptococcus gordonii* DL1 (kindly donated by P. E. Kolenbrander), *Streptococcus mutans* UA159 (kindly donated by M. C. Peters, University of Michigan, School of Dentistry, Ann Arbor, MI, USA), *Streptococcus mutans* ATCC 25175, and *Aggregatibacter actinomycetemcomitans* Y4 were grown on Brain Heart Infusion agar (BHI, Difco, Sparks, MD, USA) and cultured in BHI broth media. *Actinomyces odontolyticus* ATCC 17982, *Prevotella intermedia* (clinical isolate, kindly donated by W. Loesche, University of Michigan, School of Dentistry, Ann Arbor, MI, USA), *Fusobacterium nucleatum* ATCC 25586, were grown on Fastidious Anaerobe Agar (Acumedia, Lansing, MI, USA) containing 5% sheep blood and cultured in BHI broth media supplemented with hemin (5 μg/ml) and Vitamin K (1 μg/ml). *Treponema denticola* ATCC 35405 was grown in TYGVS broth (Ohta et al., 1986; Fenno, 2005). All bacterial species mentioned above were incubated at 37°C under appropriate atmospheric conditions in either an anaerobic chamber (Coy, Grass Lake, MI, USA) or a 5% CO_2_ incubator. For each strain, a single colony was inoculated into 5 ml of culture medium (as indicated above) and incubated to exponential growth phase. For use in experiments, the optical density at 600 nm (OD) of each culture was adjusted approximately to 0.15 to correspond to a bacterial concentration of 10^9^ CFU/ml in culture medium.

### MIC and MBC of Planktonic Oral Bacteria

The minimum inhibitory concentrations (MICs) and minimum bactericidal concentrations (MBCs) of nisin against oral Gram-positive and Gram-negative bacterial strains were determined using the Clinical and Laboratory Standards Institute (CLSI) standards with slight modifications as described below (Wikler, 2006; Wiegand et al., 2008). A total volume of 200 μl with different concentrations of nisin and the bacterial culture suspended in BHI or TYGVS broth medium was added to a 96-well microplate (Costar, Corning Inc., NY, USA). Of the total volume, each well contained 150 μl of bacterial culture and 50 μl of nisin. As mentioned above, the initial optical density of the bacterial cultures were calibrated approximately to 0.15 (OD_600_) to achieve 10^9^ CFU/ml. The final working concentrations of nisin were 0.1, 0.25, 0.5, 1, 2.5, 5, 10, 15, 25, 50, 100, 200 μg/ml. The microplates were incubated at 37°C for 24 h under aerobic or anaerobic conditions according to the bacterial growth requirements. *T. denticola* was cultured under anaerobic conditions in TYVGS media up to 6 days to determine the MIC. The determined MIC was the lowest concentration of nisin that inhibited the visible growth of bacteria compared to the zero time point, indicated by an increase (≤0.05) in optical density (OD_600_). For determination of MBC, 10 μl of these bacterial samples were removed from wells that had bacterial concentrations equivalent to and higher than the MIC, and inoculated on appropriate agar plates or in TYGVS broth medium. The MBC was defined as the lowest concentration of nisin that killed at least 99.9% of the bacteria in a given time.

### Human Saliva Collection and Preparation: Multi-Species Inoculum and Nutrient Source

A saliva collection protocol was reviewed and given “not regulated status” by the University of Michigan Institutional Review Board (IRB) for Human Subject Research (HUM00095026). This protocol has been used previously (Nance et al., 2013; Samarian et al., 2014). Briefly, for the collection of pooled human whole saliva, the protocol required that at least six healthy individuals donate saliva. These individuals had not consumed any food or beverages besides water during the 2 h prior to donation. All donors were non-smokers and had not been prescribed antibiotics for the preceding three months. Collected saliva was prepared for one of two purposes: to be used as a cell-containing saliva (CCS) inoculum or to be used as a cell-free saliva (CFS) nutrient source for biofilm growth. CCS was prepared by mixing native, pooled saliva with glycerol in a 75%/25% ratio, respectively, and then separated into 3 ml aliquots for storage at -80°C. CFS was prepared by adding 2.5 mM dithiothreitol (DTT) to the saliva then allowing it to stand for 10 min on ice followed by centrifugation at 17000 × *g* for 30 min. The resulting supernatant was mixed with distilled water to a final concentration of 25% and filter sterilized through 0.2-μm syringe filter with a cellulose polyethersulfone membrane (VWR, Radnor, PA, USA). Aliquots of 30 ml were stored at -80°C.

### Effect of Nisin on Biofilm Development in a Static Model System

Twenty-four well glass bottom sensoplates (Greiner Bio-One, Monroe, NC, USA) were used for the static model system (Kolderman et al., 2015). Fifteen microliter of CCS was inoculated into each well with 1.5 ml of CFS with or without nisin (0.5–50 μg/ml). Upon inoculation, the biofilms were incubated for 20–22 h at 37°C for overnight growth. Following overnight incubation, the CFS that remained in each well was aspirated. All wells were then washed with 500 μl of phosphate buffered saline solution (PBS) three times. Following the wash, PBS in each well was aspirated, and biofilms were stained using formulated BacLight LIVE/DEAD bacterial viability staining solution (Invitrogen, Carlsbad, CA, USA) or Syto-9 nucleic acid stain (Invitrogen, Carlsbad, CA, USA) prepared according to the manufacturer’s instructions. Syto-9 stain was used to quantify the total DNA content of the biofilm. Two hundred and fifty microliter of LIVE/DEAD or 250 μl of Syto-9 solutions were added to each well and incubated for 45 min at room temperature and washed with 500 μl of PBS three times. For capturing three-dimensional (3D) images of biofilms, confocal laser scanning microscopy was used as described below. The total DNA content of the biofilm was measured using a Victor X3 2030 Multi-label reader (PerkinElmer, Waltham, MA, USA) by detecting fluorescence intensity at 530 nm.

### Effect of Nisin on Biofilm Development in a Controlled Flow Microfluidic System

Forty-eight-well Bioflux microfluidic plates (Fluxion, San Francisco, CA, USA) in conjunction with the Bioflux 200 system (Fluxion, San Francisco, CA, USA) were used for the microfluidic model system (Nance et al., 2013; Samarian et al., 2014). Prior to adding the CCS, the microfluidic channels were first pre-treated with CFS for cell attachment, salivary pellicle formation, and biofilm development. One hundred microliter of CFS was added to each outlet well, then flowed toward the inlet well at 1.0 dyn/cm^2^ (equivalent to the flow rate of 93 μl/h, corresponding to a shear of 100 s^-1^ through the channel) for 2 min at room temperature. Flow was then stopped and the plate was incubated at room temperature for 20 min. Once pre-treatment incubation was completed, the CFS remaining in each outlet well was transferred to the corresponding inlet well, then 100 μl of CCS was added to each outlet well. To introduce bacterial cells into the plate channel for viewing biofilm growth, the CCS was flowed from outlet to inlet wells at 1.0 dyn/cm^2^ for 6 s at 37°C. Upon inoculation of bacterial cells into the growth/viewing channels, cell seeding was confirmed visually with a Nikon Eclipse TCS-100 inverted light microscope equipped with a 20X0.40 NA PH1 infinity-corrected objective. The plate was then set to incubate at 37°C for 40 min to allow cell adherence and initial growth of the biofilm prior to the nutrient flow. Following incubation, CCS was aspirated from each outlet well and 800 μl of CFS with or without nisin (0.5–50 μg/ml) was added to each inlet well. Plates were then incubated at 37°C for 20–22 h with controlled flow of 0.2 dyn/cm^2^ (flow rate of 19 μl/h, corresponding to a shear of 20 s^-1^) for overnight biofilm growth. Following overnight incubation, CFS in each inlet and outlet well was aspirated. One hundred microliter of PBS was added to each inlet well and flowed at room temperature for 20 min at 0.2 dyn/cm^2^ to remove remaining treatment solution. Following the wash, PBS in the inlet wells was aspirated, and biofilms were stained using formulated BacLight LIVE/DEAD bacterial viability staining solution (Invitrogen, Carlsbad, CA, USA). One-hundred microliter of formulated BacLight solution was added to each inlet wells and flowed at 0.2 dyn/cm^2^ for 45 min at room temperature to allow staining of the biofilms. Once the staining was complete, the remaining solutions in the inlet wells were aspirated and 100 μl of PBS was added to each inlet well and flowed at 0.2 dyn/cm^2^ for 20 min at room temperature to remove any excess stain in the Bioflux channels. For capturing 3D images of biofilms, confocal laser scanning microscope was used as described below.

### Disruption of Pre-Formed Biofilms by Nisin

To study the effect of nisin on pre-formed biofilms and to precisely control the exposure times, biofilms were inoculated and grown in 24 well Sensoplates (Greiner Bio-One, Monroe, NC, USA) as described above using CCS and CFS for 20–22 h at 37°C. Following overnight growth, the biofilms were treated with nisin (10, 50 μg/ml) with short exposure times (1, 5, 10 min). Following the treatment of pre-formed biofilms with or without nisin in CFS, all wells were washed with PBS three times. The same biofilm staining protocol was followed as described above.

### Confocal Laser Scanning Microscopy and Quantitative Analysis of Biofilms

After nisin treatment, biofilms were imaged using Leica confocal laser scanning microscopy (CLSM, SPE, Leica, IL, USA) with a 40X1.25 NA HCX PL APO infinity-corrected oil or a HCX PL APO 40X/0.85 CORR CS objective. All biofilms were stained with the BacLight Live/Dead Bacterial Viability kit (Invitrogen, Carlsbad, MA, USA), which contains the nucleic acid stains Syto-9 (green signal) and propidium iodide (red signal) as described above. Once the microscopy images were taken, biofilms were rendered as 3D structures with Imaris (Bitplane, Zurich, Switzerland) computer imaging software. Image stacks were treated equally and the signal intensity of rendered 3D biofilm structures were confirmed using histograms generated in Imaris. Imaris allowed for the visualization of biofilm architecture in three dimensions, penetration of nisin into biofilms (inferred by the extent and degree of the red signal), and the preparation of 3D files for the quantification of biofilm structure using the computer software program Comstat2. For detailed biofilm analysis, Comstat2 was used to determine the biofilm biovolume (total amount of space/biomass occupied by a biofilm), average thickness (thickness of each biofilm extending from the bottom to the top of the growth/viewing channel surface), and roughness (a measure of heterogeneity in biofilm architecture). The degree of killing, based on green (Syto-9; live) and red (PI; dead/damaged) pixel intensity for every pixel in all 3D planes were evaluated using ImageJ (National Institutes of Health). The percentages of live to dead/damaged cells was determined by first multiplying the total number of pixels by the level of intensity (0–255) and then summing the total value for both the LIVE and DEAD signals from each image stack recorded. All renderings and quantification analyses were performed on a dedicated laptop computer equipped with an Intel Core i5 CPU with 8 GB RAM, 64-bit operating system (MSI Computer Corp., Industry, CA, USA).

### Cell Culture

A direct cell outgrowth technique was used to obtain primary periodontal ligament (PDL) cells and gingival fibroblast (GF) cells as previously described (Scanlon et al., 2011). These cells were maintained in minimum essential medium alpha (α-MEM) supplemented with 10% fetal bovine serum (FBS), 1% penicillin/streptomycin, and 1% fungizone (Gibco, Life Technologies, Grand Island, NY, USA). Passage 3 or 4 PDL and GF cells were used for experiments. Primary human oral keratinocytes (OK) were purchased from Science Cell Research Laboratories (Carlsbad, CA, USA) and maintained in keratinocyte growth medium (KGM) supplemented with OK growth supplements (OKGS) and 1% penicillin/streptomycin. Passage 1 or 2 OK cells were used for experiments. Osteoblast-like cells were purchased from American Type Culture Collection (MG63; Manassas, VA, USA) and maintained in α-MEM supplemented with 10% FBS, 1% penicillin/streptomycin, and 1% fungizone. Passage 2 to 4 MG63 cells were used for experiments.

### Apoptosis Staining and Microscopy

To assess the effects of nisin on cell viability and nuclear morphology, an acridine orange/ethidium bromide (AO/EB) staining assay was used as described previously (Ribble et al., 2005; Kasibhatla et al., 2006). AO (Acros Organics, Geel, Belgium) and EB (Bio-Rad laboratories, Berkeley, CA, USA) are fluorescent DNA binding dyes that can be combined to assess apoptotic cellular changes and cell membrane integrity. Using a 96-well microplate (Thermo Scientific, Waltham, MA, USA), cells (GF, PDL, OK, and MG63 cells) were plated at 2 × 10^4^ cells/cm^2^ and allowed to adhere and spread overnight. After 24 h, cell cultures were exposed to nisin (final concentrations: 1, 10, 100, 200, 400, 800 μg/ml) or left untreated and incubated for a following 24–48 h at 37°C. Following each treatment period, the cells were stained with the AO/EB dye solution for one minute under gentle agitation. The AO/EB dye reagent was comprised of 100 μg/ml of ethidium bromide and 100 μg/ml of acridine orange in PBS. The dye solution was removed and the cells were viewed and imaged using an epifluorescence microscope (Eclipse 50i, Nikon, Melville, NY, USA). For cell counting, each well was divided into two halves where a minimum of 150 cells was counted per half at 4× magnification. Cell viability, apoptosis, and necrosis were assessed as described previously (Ribble et al., 2005; Kasibhatla et al., 2006). Experiments were performed in triplicate.

### Cell Proliferation Assay

The effect of nisin on cell proliferation was assessed by measuring the level of intracellular lactate dehydrogenase (LDH) activity using the Cell Counting Kit-8 (Dojindo, Kumamoto, Japan) per manufacturer’s recommendations. Using a 96-well microplate (Thermo Scientific, Waltham, MA, USA), the cells (GF, PDL, OK, and MG63 cells) were plated at 2 × 10^4^ cells/cm^2^ and allowed to adhere and spread overnight. The subsequent day, the cells were treated with different concentrations of nisin (10–800 μg/ml) for 24–48 h. After 24 or 48 h, 10 μl of the CCK-8 solution was added to each well and incubated at 37°C for 3 h, then absorbance was measured at 450 nm using a microplate reader (Spectra Max M2, Molecular Devices, Sunnyvale, CA, USA) to determine the LDH activity. Cell proliferation was evaluated based on the LDH activity measured as absorbance values at 450 nm.

### Statistical Analysis

Values are expressed as mean values ± SD. Independent *t*-tests were used to compare the control (nisin-free) with nisin-treated samples. The difference between the viability, apoptosis, and necrosis was analyzed by ANOVA, using Dunnett’s method. Individual *P*-values for each data set are indicated either individually or as a group in each figure. All experiments were repeated at least three times. Values of *P* < 0.05 were considered significant, and *P* < 0.01 were considered highly significant.

## Results

### Antimicrobial Activity of Nisin on Oral Biofilm Colonizers

**FIGURE 1:**
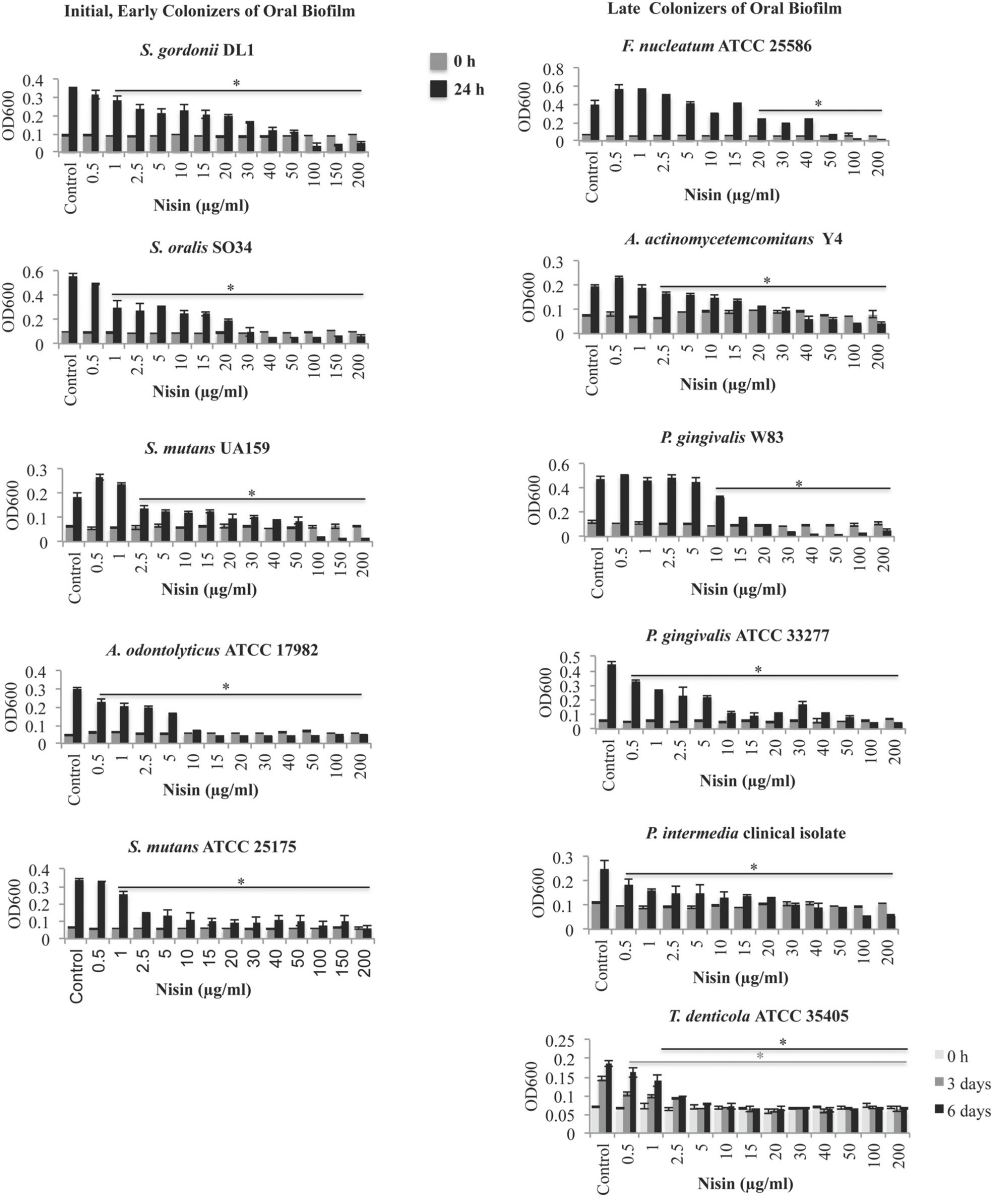
**Nisin inhibits the growth of cariogenic and periodontal pathogens.** Using a broth dilution method, *S. gordonii* DL1, *S. oralis* SO34, *S. mutans* UA159, *A. odontolyticus* ATCC 17982, *S. mutans* ATCC 25175, *F. nucleatum* ATCC 25586, *A. actinomycetemcomitans* Y4, *P. gingivalis* W83, *P. gingivalis* ATCC *33277, P. intermedia* clinical isolate *and T. denticola* ATCC 35405 was cultured with or without nisin (0.1–200 μg/ml) on a microplate for 24 h at 37°C, under aerobic or anaerobic conditions. The determined MIC was the lowest concentration of nisin that inhibited the visible growth (≤0.05 increase in OD_600_ after 24 h growth) of the inoculated bacteria. ^∗^*P* < 0.05: significant differences from the control (nisin-free).

The MICs and MBCs of nisin on oral bacterial species are listed in **Table 1**. The MIC and MBC of nisin ranged from 2.5 to 50 μg/ml and 15 to 200 μg/ml, respectively, for early, middle, and later colonizers of dental plaque (**Table 1**). *S. mutans*, a cariogenic Gram-positive bacteria, showed a 1.5- to 3-fold higher sensitivity to nisin when compared to the two commensal organisms *S. gordonii* and *S. oralis* (**Figure 1**; **Table 1**). *F. nucleatum*, a Gram-negative bacteria known for its prominent role in coaggregation with both early and later colonizers of dental plaque showed the least susceptibility amongst the tested species with a MIC of 50 μg/ml (**Figure 1**; **Table 1**). In addition, nisin exerted antimicrobial activities against the known Gram-negative periodontal pathogens, including *P. gingivalis*, *P. intermedia, A. actinomycetemcomitans*, and *T. denticola* (**Figure 1**; **Table 1**) at nisin concentrations between 2.5 and 20 μg/ml. Amongst the later colonizers of oral biofilms, *T. denticola* exhibited the highest sensitivity to nisin with a MIC of 2.5 μg/ml and MBC of 15 μg/ml.

**Table 1:** Minimum inhibitory concentrations (MICs) and minimum bactericidal concentrations (MBCs) of planktonic oral pathogens.

Oral microorganisms	MIC	MBC	MBC/MIC
**Gram positive – Early, middle colonizers of oral biofilm**			
*Streptococcus gordonii* DL1	40 μg/ml	150 μg/ml	3.75
*S. oralis* SO34	30 μg/ml	150 μg/ml	5
*S. mutans* UA159	20 μg/ml	100 μg/ml	5
*Actinomyces odontolyticus* ATCC 17982	10 μg/ml	30 μg/ml	3
*S. mutans* ATCC 25175	10 μg/ml	200 μg/ml	20
**Gram negative – Late colonizers of oral biofilm**			
*Fusobacterium nucleatum* ATCC 25586	50 μg/ml	150 μg/ml	3
*Aggregatibacter actinomycetemcomitans* Y4	15 μg/ml	100 μg/ml	6.67
*Porphyromonas gingivalis* W83	20 μg/ml	100 μg/ml	5
*Porphyromonas gingivalis* ATCC 33277	15 μg/ml	100 μg/ml	6.67
*Prevotella intermedia* clinical isolate	10 μg/ml	150 μg/ml	15
*Treponema denticola* ATCC 35405	2.5 μg/ml	15 μg/ml	6

MBC/MIC ratio of >4 indicates that nisin has a bacteriostatic effect. MBC/MIC ratio of <4 indicates that nisin has a bactericidal effect (Pankey and Sabath, 2004).

### The Anti-biofilm Effects of Nisin on Formation of Multi-Species Biofilms

**FIGURE 2:**
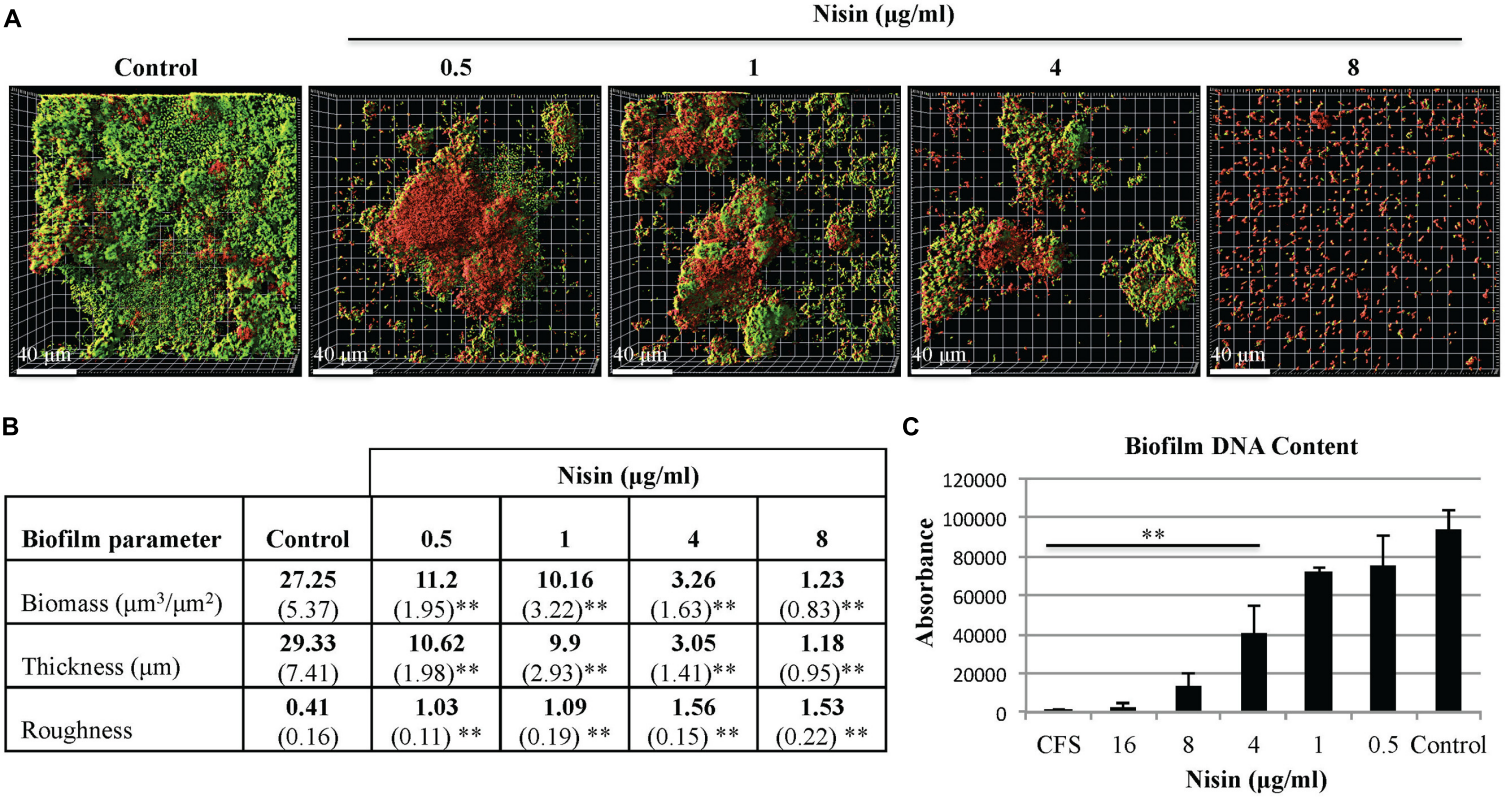
**Nisin inhibits the formation of multi-species biofilms in a static model system.** Cell-containing saliva (CCS) was inoculated in filter sterilized cell-free saliva (CFS) for 20–22 h at 37°C with or without nisin. **(A)** Confocal microscopy images are represented in the x–y plane. A green signal indicates viable live cells (Syto 9), a red signal indicates damaged/dead cells (propidium iodide), **(B)** Biofilm biomass, thickness, and roughness [mean (SD)] were derived from imaging of at least three separate wells (experiments), **(C)** DNA content of the biofilms was quantified by absorption spectroscopy at fluorescence intensity of 530 nm. ^∗^*P* < 0.05 and ^∗∗^*P* < 0.01: significant differences from the control (nisin-free).

Saliva derived multi-species biofilms were grown with or without nisin using static and microfluidic model systems. Under the static growth condition, early signs of biofilm membrane damage was observed at nisin concentrations ≥0.5 μg/ml (**Figure 2A**). From confocal microscopy imaging and quantitative analysis, the biofilm growth was significantly reduced at nisin concentrations ≥4 μg/ml (**Figure 2C**). In the static system, the average biofilm biomass and thickness of the control biofilms were 27.25 μm^3^/μm^2^ and 29.33 μm (**Figure 2B**). The average biofilm biomass and thickness of the nisin treated biofilms at 4 μg/ml were 3.26 μm^3^/μm^2^ and 3.05 μm (**Figure 2B**). At 8 μg/ml nisin, the biofilm growth and architecture was abrogated when compared to the control conditions (**Figure 2A**).

**FIGURE 3:**
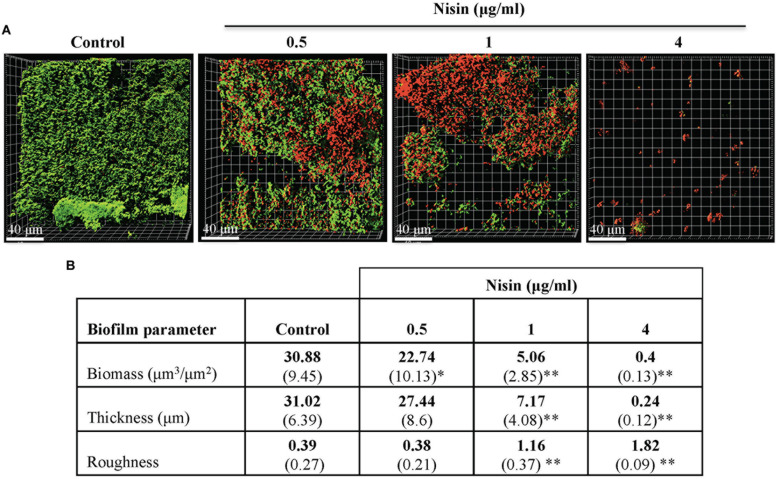
**Nisin inhibits the formation of multi-species biofilms in a Bioflux controlled flow microfluidic model system.** CCS was added, then fed filter sterilized CFS for 20–22 h at 37°C with or without nisin. **(A)** Confocal microscopy images are represented in the x–y plane. A green signal indicates viable live cells (Syto 9) and a red signal indicates damaged/dead cells (propidium iodide). **(B)** Biofilm biomass, thickness, and roughness [mean (SD)] were derived from imaging of at least three separate channels (experiments). ^∗^*P* < 0.05 and ^∗∗^*P* < 0.01: significant differences from the control (nisin-free).

Under controlled flow microfluidic growth conditions, the anti-biofilm effects of nisin were exerted at concentrations ≥0.5 μg/ml (**Figure 3A**). The average biofilm biomass and thickness of the control biofilms were 30.88 μm^3^/μm^2^ and 31.02 μm (**Figure 3B**). The average biofilm biomass and thickness of the nisin treated biofilms at 1 μg/ml were 5.06 μm^3^/μm^2^ and 7.17 μm (**Figure 3B**). The formation of biofilms was absent at 4 μg/ml (**Figures 3A,B**).

When evaluated under both biofilm model systems, biofilm biomass and thickness were significantly reduced when the biofilms were grown in presence of nisin at concentrations ≥1 μg/ml (**Figures 2B** and **3B**). The roughness coefficient values of the biofilms increased in a dose-dependent manner, suggesting an increase in heterogeneity within the biofilm architecture due to membrane associated structural damages. Collectively, our data suggest that nisin concentrations >8 μg/ml can inhibit the formation of saliva derived multi-species biofilms.

### The Anti-biofilm Effects of Nisin on Preformed Multi-Species Biofilms

**FIGURE 4:**
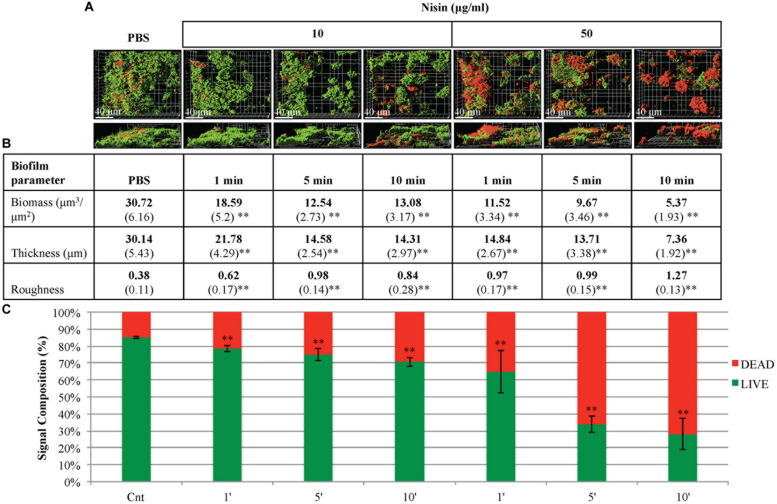
**Nisin disrupts the maintenance of three-dimensional architecture of pre-formed biofilms.** CCS was inoculated in filter sterilized CFS for 20–22 h at 37°C and treated with phosphate buffered saline solution (PBS) solution (control) or nisin at different concentrations and incubation times. **(A)** Confocal microscopy images are represented in the x–y and x–y–z plane. A green signal indicates viable live cells (Syto 9) and a red signal indicates damaged/dead cells (propidium iodide). **(B)** Biofilm biomass, thickness, and roughness [mean (SD)] were derived from imaging of at least three separate wells (experiments). **(C)** An average percentage signal from the biofilms was determined by the Live/viable signal (green) and the Dead/damaged signal (red) in relation to the total signal captured for both. ^∗^*P* < 0.05 and ^∗∗^*P* < 0.01: significant differences from the control (nisin-free).

Saliva derived multi-species biofilms formed overnight (20–22 h) under static conditions, then treated with two different concentrations of nisin (10, 50 μg/ml). The biofilms were treated with nisin for short exposure times (1, 5, and 10 min). At both concentrations and at all three exposure times, the 3D rendered confocal microscopy images of biofilms demonstrated structural damage and dissociation within the biofilm architecture (**Figure 4A**). When compared to the control biofilms treated with PBS, nisin treated biofilms exhibited reduced biofilm biomass and thickness (**Figure 4B**). This reduction in the biomass and thickness of the biofilms occurred in a dose- and time-dependent manner. At a nisin concentration of 10 μg/ml, the viability of the biofilms was only slightly reduced for all exposure times (although statistically significant). However, at 50 μg/ml, much higher biofilm killing was observed at 5 and 10 min exposure times, as indicated by the confocal microscopy imaging and Live/Dead signal quantification of the biofilms (**Figures 4A,C**). In addition, the roughness coefficient values of the nisin treated biofilms were significantly increased compared to the control biofilms treated with PBS. The increase in the roughness coefficient values occurred in a time-dependent manner at 50 μg/ml, but it did not follow a specific time-dependent trend with 10 μg/ml.

### The Effects of Nisin on Viability of Human Cells Relevant to the Oral Cavity

**FIGURE 5:**
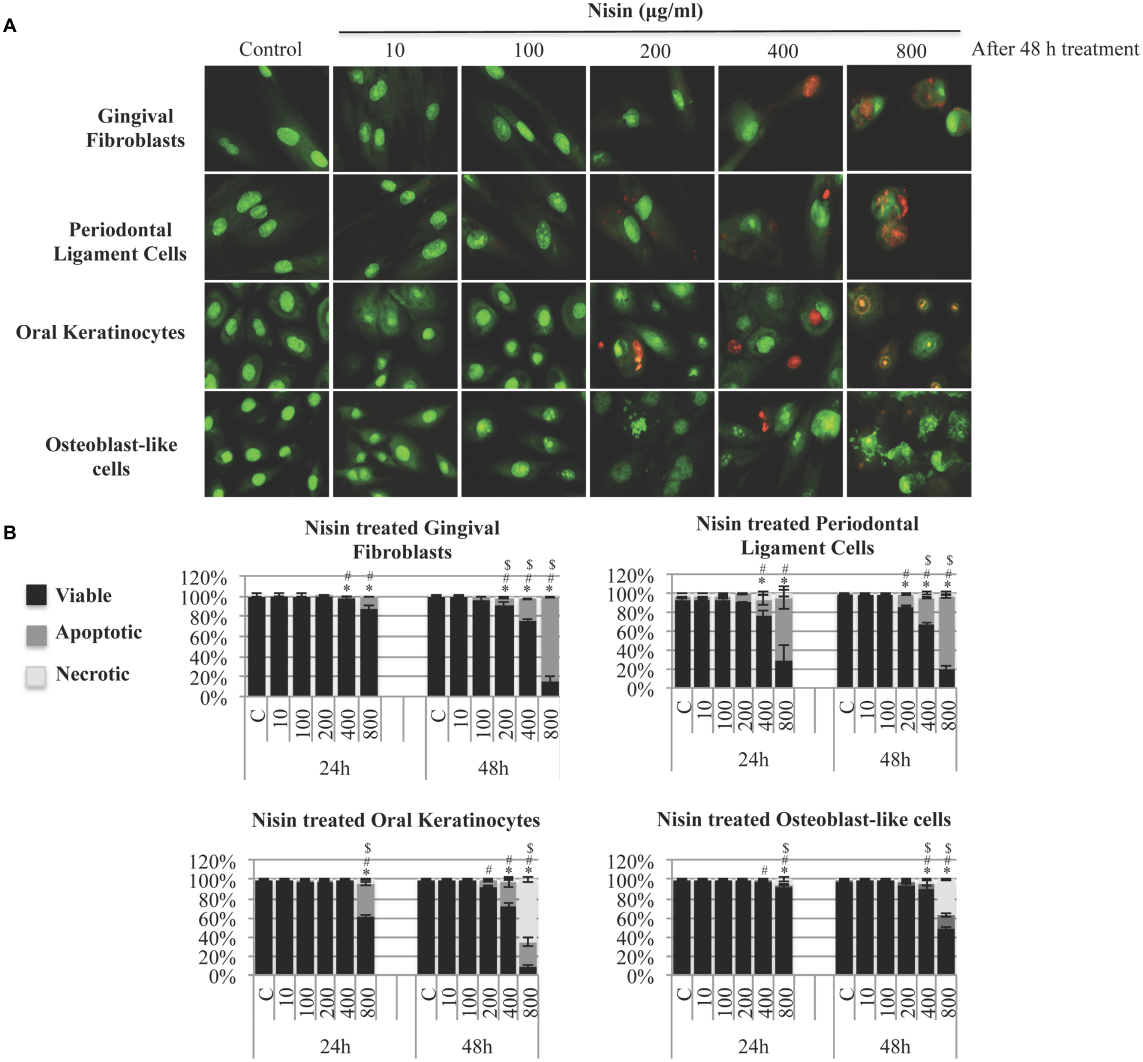
**Nisin has minimal cytotoxicity to human cells relevant to the oral cavity.** Primary human gingival fibroblast (GF) cells, periodontal ligament (PDL) cells, oral keratinocyte (OK) cells, and osteoblast-like cells were incubated with nisin (1–800 μg/ml) for 24–48 h on a 96-well microplate at 37°C. Cells were then stained with acridine-orange/ethidium-bromide (AO/EB) to evaluate cell viability, apoptosis and necrosis using epifluorescence microscopy. **(A)** Images of the cells after 48 h incubation period with nisin at different concentrations. **(B)** The cytotoxicity of nisin was quantified by counting viable, apoptotic and necrotic cells and expressed as bar graphs with heights representing mean % and error bars representing SD. AO (green) stained cells with intact membrane integrity. Early apoptotic cells stained green but contained bright green dots in the nuclei due to chromatin condensation and nuclear fragmentation. Late apoptotic cells stained with EB (orange) with apoptotic phenotypes. Necrotic cells stained orange but the nuclear morphology resembled the viable cells with absence of chromatin condensations. Mean values were calculated with SD. ^∗^,#,$ represent *P* < 0.05: significant differences from the control (nisin free) for viability, apoptosis and necrosis, respectively.

The effect of nisin on cell viability was assessed in human cells that are relevant to the oral cavity. After a 24 h treatment, GF, PDL, OK, and MG63 cells were highly tolerant to nisin treatments (**Figure 5B**). With nisin treatments of up to 400 μg/ml, GF, PDL, OK, and MG63 cells exhibited normal cell viability levels (>95%) and cell phenotypes with minimal apoptotic characteristics. After a 48 h treatment, GF, PDL, and OK cells exhibited normal cell viability with nisin up to 200 μg/ml. Osteoblast-like MG63 cells exhibited the highest tolerance against nisin (**Figure 5B**). At antimicrobial and anti-biofilm concentrations (<100 μg/ml), nisin treated cells maintained a normal cell shape and nuclear phenotype compared to the untreated control cells. Only at concentrations ≥200 μg/ml and after 48 h, cells started showing low levels of apoptosis with chromatin condensation and nuclear fragmentation (**Figure 5A**).

### The Effects of Nisin on Cell Proliferation

**FIGURE 6:**
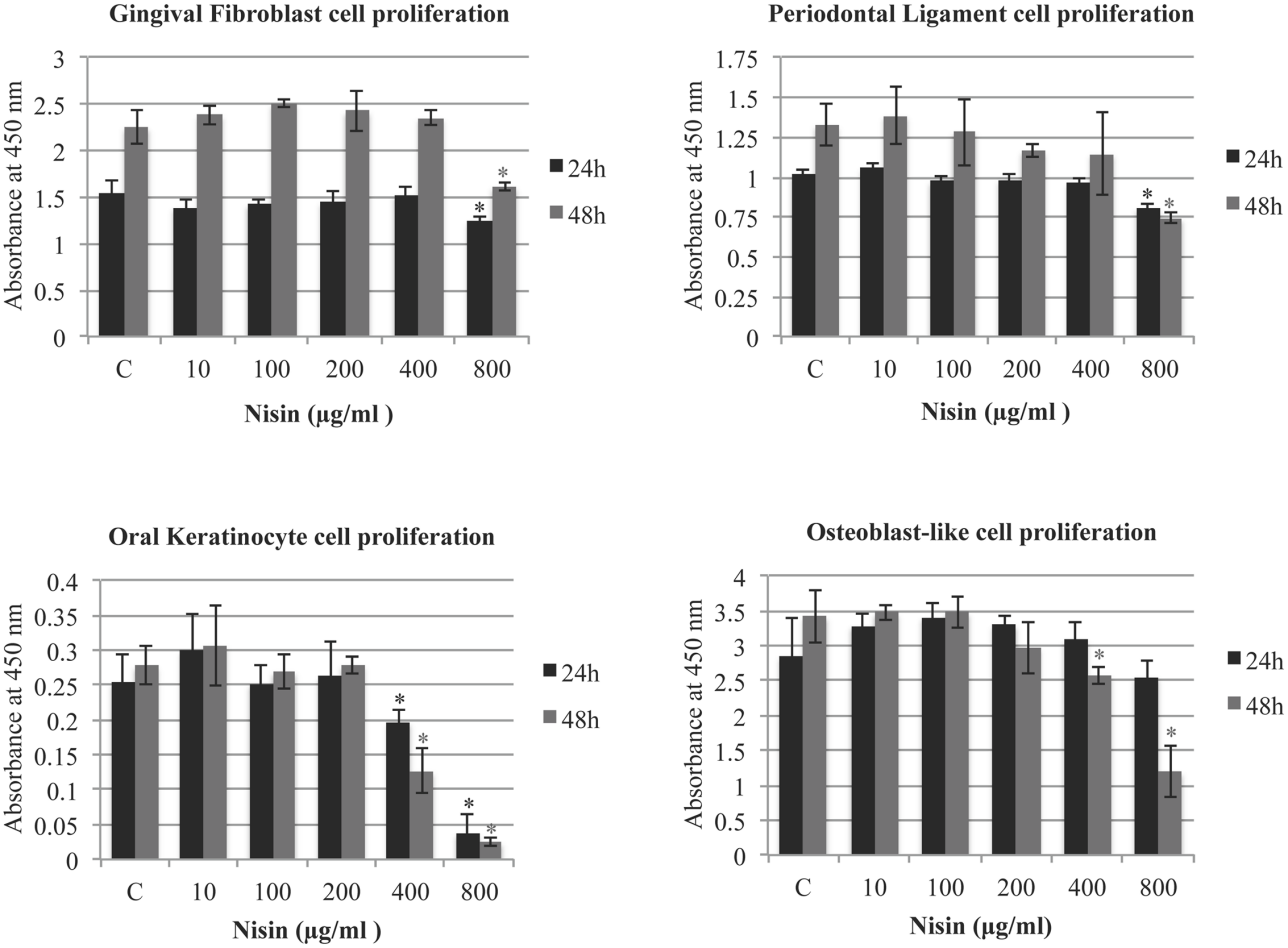
**Nisin does not effect cell proliferation of human cells.** The effect of nisin on cell proliferation was assessed using a Cell Counting Kit-8 measuring the lactate dehydrogenase (LDH) activities in cells. Using a 96-well microplate, GFs, PDL cells, OKs, and osteoblast-like cells were plated at 2 × 10^4^ cells/cm^2^ and incubated for 24–48 h in the presence or absence of nisin (1–800 μg/ml). At 24 and 48 h time points, LDH levels were measured using absorption spectroscopy at 450 nm. Mean values were calculated with SD. ^∗^*P* < 0.05: significant differences from the control (nisin-free).

The effect of nisin on cell proliferation was assessed by measuring the innate LDH activity of the cells (GF, PDL, OK, MG63). After 24 h with treatment of up to 800 μg/ml of nisin, GF, PDL and MG63 cells exhibited normal cell proliferation compared to the control (**Figure 6**). In control experiments, nisin (10–800 μg/ml) incubated in media alone exhibited negligible levels of LDH activity (data no shown). Only OK cells exhibited reduced cell proliferation after a 24 h nisin treatment at concentrations >400 μg/ml. In addition, GF and PDL exhibited normal cell proliferation with nisin concentrations up to 800 μg/ml, and OK and MG63 exhibited normal cell proliferation with nisin concentrations up to 400 μg/ml (**Figure 6**). At 800 μg/ml, OK cells were unable to attach to the cell surface and proliferate. At antimicrobial and anti-biofilm concentrations (<100 μg/ml), all cells exhibited normal cell attachment and proliferation.

## Discussion

The data presented here demonstrate the potential for nisin as an antimicrobial and anti-biofilm agent against oral pathogens. The peptide structure of nisin is characterized by the presence of five intra-molecular rings formed by the thioether amino acids lanthionine and 3-methyllanthionine (Wiedemann et al., 2001). Due to its unique chemical features, it has been hypothesized that nisin has various modes of antimicrobial action (Peschel and Sahl, 2006) and exerts multiple antimicrobial activities based on the interaction with multiple cellular targets (Bierbaum and Sahl, 1985; Chan et al., 1996; Pag and Sahl, 2002). However, when used in a clinical setting, there is potential risk of developing nisin resistance. There have been a few examples of lantibiotic resistance noted in laboratory settings, where certain bacteria have been reported to possess innate anti-lantibiotic mechanisms. For example, nisinase is a dehydropeptide reductase that can inactivate nisin (de Freire Bastos et al., 2014; Draper et al., 2015). Nisinase activity has been associated with *Lactobacillus plantarum* (Kooy, 1952), *Streptococcus thermophilus* (Alifax and Chevalier, 1962), *Clostridium botulinum* (Rayman et al., 1983), *Lactococcus lactis* sub-species *cremoris*, *Enterococcus faecalis*, and *Staphylococcus aureus* (Carlson and Bauer, 1957). Thus, future characterization of specific genetic or protein components that may contribute to nisin resistance is needed to understand any potential resistance issues in a clinical setting.

Previously, studies have implicated that nisin has broad-spectrum activity against Gram-positive bacteria with limited potency on Gram-negative bacteria (Delves-Broughton et al., 1996; Severina et al., 1998; Cleveland et al., 2001). Our findings demonstrate that the high purity form of nisin Z (nisin ZP, >95% purity) exhibits antimicrobial activity against both Gram-positive and Gram-negative oral bacteria. In comparison to recent studies that used a low content nisin (Tong et al., 2010; Corbin et al., 2011), our data suggests that nisin ZP (>95% purity) is much more potent than low content nisin A (2.5% purity) in inhibiting cariogenic oral bacteria and oral biofilms. These earlier studies utilized low purity nisin, which is generally less soluble than the high purity form. In addition, when considering the preparation of nisin solutions, nisin A (2.5% purity) requires approximately pH 2 whereas nisin ZP can be prepared at more neutral pH conditions (Pag and Sahl, 2002). Nisin ZP can be easily prepared in water at pH 7 as a colorless, odorless, and tasteless solution. Hence, the utilization of high purity nisin ZP as a potential oral anti-microbial rinse has promising features.

Commensal oral bacteria, such as S. *gordonii* and *S. oralis*, exhibited lower sensitivity to nisin than the pathogenic species *S. mutans* (**Table 1**). From the single species experiments, the most interesting data resulted from the Gram-negative anaerobes. Periodontal disease is strongly associated with pathogenic bacteria such as *P. gingivalis*, *P. intermedia, A. actinomycetemcomitans*, and *T. denticola*. These anaerobic pathogens are believed to gain access to the periodontal tissues and thereby mediate tissue damage by a complex array of host–pathogen interactions, including modulation of inflammatory host response mechanisms (Haffajee and Socransky, 1994; Pihlstrom et al., 2005; Darveau, 2009). In our study, nisin at low concentrations <20 μg/ml inhibited the growth of these periodontal pathogens (**Table 1**). Amongst these pathogens, *T. denticola* was highly susceptible to nisin treatment, requiring only 2.5 μg/ml to inhibit its growth up to 6 days (**Figure 1**). *T. denticola* comprises up to 30% of the microbiota in diseased gingival pockets and is associated with tissue and bone destructive mechanisms in periodontitis (Baehni et al., 1992; Riviere et al., 1992; Gopalsami et al., 1993; Choi et al., 2003). Thus, our findings suggest that nisin can inhibit the growth of both Gram-positive and Gram-negative disease-associated oral bacteria.

Dental plaque is a complex of multi-species biofilm community (Marsh, 2010). Collectively, the contained species can withstand the constantly changing conditions of the oral cavity and can be highly resistant to the treatment of antimicrobial agents (Jakubovics and Kolenbrander, 2010). Since these biofilms can rapidly form and mature to develop additional pathogenic traits, anti-biofilm agents that inhibit the formation of and disperse established biofilms would benefit the prevention and treatment of oral diseases (Marsh, 2010). Using biologically relevant human saliva as the multi-species biofilm inoculum and growth media, our results demonstrated that nisin exerted anti-biofilm properties. Overnight incubation of nisin within saliva caused substantial inhibition of biofilm formation. Specifically, nisin concentrations ≥0.5 and 1 μg/ml caused a significant reduction in biofilm biomass and thickness of biofilms developed in static and microfluidic systems, respectively (**Figures 2B** and **3B**). The resulting biofilms were highly disintegrated and lacked coaggregative behavior expressed in the control untreated biofilms (**Figures 2A** and **3A**).

Similar to preventing biofilm development, the anti-biofilm properties of nisin against pre-formed biofilms occurred in a dose- and time-dependent manner. As inferred by observed changes in biofilm architecture, biofilms seemingly dispersed and sloughed into smaller aggregates after a 1 min exposure to 10 and 50 μg/ml of nisin. (**Figure 4A**). At 50 μg/ml and after a 5 min exposure, biofilms exhibited cell death, and membrane damage indicated by the Live/Dead signal quantification (**Figure 4C**). However, at 10 μg/ml, significant bacterial killing was not observed regardless of the treatment time. The antimicrobial action of nisin may be either bacteriostatic or bactericidal depending on multiple factors, such as nisin concentration, bacterial concentration, physiological state of the bacteria, and the prevailing conditions (Delves-Broughton et al., 1996). Thus, our data support the premise that nisin acts as a fast-acting anti-biofilm agent with both biofilm-static and biofilm-killing properties.

Coaggregation of different bacterial species is considered critical to maintaining the stability of the architecture and species composition of dental plaque (Hojo et al., 2009; Katharios-Lanwermeyer et al., 2014). Coaggregation interactions promote the development of multi-species biofilms by enabling bacterial communication and colonization of initial, middle, and later colonizers (Kolenbrander et al., 2010). Previously, Smith et al. (1991) demonstrated that cationic antimicrobials, such as chlorhexidine digluconate and cetylpyridinium chloride, can selectively inhibit coaggregation interactions of later colonizers of dental plaque. As a cationic and a membrane acting bacteriocin, the modes of action of nisin may be to disrupt the coaggregation process needed to form a stable biofilm. Present data are suggestive that the likely role of nisin in inhibiting coaggregation may be at least in part responsible for its anti-biofilm effects. Assuming that the coaggregation process is critical for developing and maintaining the biofilm complex, studies are in progress to determine the ability of nisin to inhibit specific coaggregation interactions between oral biofilm colonizers.

After more than thirty years of use as an antiplaque agent in the dental profession, chlorhexidine is still considered the gold standard (Jones, 1997; Baehni and Takeuchi, 2003). However, frequent or long-term use of chlorhexidine, although still employed clinically, is associated with negative effects (Flötra et al., 1971; Gagari and Kabani, 1995). Previously it was reported that direct exposure to as little as 0.0025 to 0.01% of chlorhexidine can significantly affect cell morphology and cell attachment of cultured GFs (Cline and Layman, 1992). In addition, chlorhexidine is highly cytotoxic to neutrophils, human epithelial cells, PDL cells, fibroblasts and HeLa cells (Goldschmidt et al., 1977; Chang et al., 2001). Our study demonstrates that orally relevant human cells are highly tolerant to direct contact by nisin. Our data indicate that nisin at antimicrobial and anti-biofilm concentrations (<100 μg/ml) is not cytotoxic to these cells (OKs, GFs, PDL cells, and osteoblast-like cells) that play an integral role in the maintenance of healthy gingival tissues. It is essential to preserve the cellular characteristics of primary OKs, since these cells act as the first line of defense against the oral pathogens (Weinberg et al., 1998; Hans and Madaan Hans, 2014). When cells were incubated with nisin for 24–48 h, cell apoptosis was nearly absent unless treated with nisin at >200 μg/ml, which is more than 10-fold greater than the minimum concentration displaying anti-biofilm effects. In addition, all cell types exhibited normal cell attachment and proliferation when treated with up to 400 μg/ml of nisin after a 24 h incubation. Cationic antimicrobial peptides are unique in that they are hypothesized to play an important role in the immune system or to exert different effects on eukaryotic cells (Hancock and Diamond, 2000). Although bacterially secreted, nisin is known to trigger immune responses in host eukaryotic cells (Hancock and Diamond, 2000; Begde et al., 2011). As an immunogenic agent, nisin was reported to elevate the T-cell population (CD4 and CD8) while reducing the B-cell population (Pablo et al., 1999). In addition, nisin has been shown to modulate the innate immune response through the induction of chemokine synthesis and suppression of lipopolysaccharide induced pro-inflammatory cytokines, both *in vitro* and *in vivo* (Kindrachuk et al., 2013). Thus, at certain non-toxic concentrations to the oral cells, nisin could potentially aid in the induction of innate defense mechanisms to help clear the oral pathogens.

The work presented here demonstrates that nisin is a promising candidate for development as an oral therapeutic anti-biofilm agent. High purity food grade nisin (>95%) exerted anti-biofilm effects against saliva derived multi-species biofilms without causing cytotoxic effects to the human oral cells. In addition to its long history and utilization as a food preservative, nisin possesses great potential for other applications involving treating clinical bacterial infections and inhibiting biofilm growth. Further investigation of the clinical role of nisin in modulating the microbiome of the biofilm community and its immunomodulatory role in human oral cells are necessary to determine its potential as a therapeutic or prophylactic agent against oral diseases.

## Conflict of Interest Statement

The authors declare that the research was conducted in the absence of any commercial or financial relationships that could be construed as a potential conflict of interest.
